# Guanylate-Binding Protein 2 Exerts GTPase-Dependent Anti-Ectromelia Virus Effect

**DOI:** 10.3390/microorganisms11092258

**Published:** 2023-09-08

**Authors:** Zhenzhen Gao, Zejing Meng, Xiaobing He, Guohua Chen, Yongxiang Fang, Huihui Tian, Hui Zhang, Zhizhong Jing

**Affiliations:** 1 State Key Laboratory for Animal Disease Control and Prevention, Chinese Academy of Agricultural Sciences, Lanzhou 730046, China; 13321288521@163.com (Z.G.); hexiaobing@caas.cn (X.H.); chengguohua78@163.com (G.C.); yongxiangf@163.com (Y.F.); tianhuihui2208@163.com (H.T.); 17300223655@163.com (H.Z.); 2 Ministry of Agriculture Key Laboratory of Veterinary Public Health, Chinese Academy of Agricultural Sciences, Lanzhou 730046, China; 3 Lanzhou Veterinary Research Institute, Chinese Academy of Agricultural Sciences, Lanzhou 730046, China; 4 School of Public Health, Lanzhou University, Lanzhou 730000, China; mengzejing2022@163.com

**Keywords:** guanylate binding protein (GBP), IFN-stimulated genes (ISGs), ectromelia virus (ECTV), GTPase

## Abstract

Guanylate-binding proteins (GBPs) are highly expressed interferon-stimulated genes (ISGs) that play significant roles in protecting against invading pathogens. Although their functions in response to RNA viruses have been extensively investigated, there is limited information available regarding their role in DNA viruses, particularly poxviruses. Ectromelia virus (ECTV), a member of the orthopoxvirus genus, is a large double-stranded DNA virus closely related to the monkeypox virus and variola virus. It has been intensively studied as a highly effective model virus. According to the study, GBP2 overexpression suppresses ECTV replication in a dose-dependent manner, while GBP2 knockdown promotes ECTV infection. Additionally, it was discovered that GBP2 primarily functions through its N-terminal GTPase activity, and the inhibitory effect of GBP2 was disrupted in the GTP-binding-impaired mutant GBP2*^K51A^*. This study is the first to demonstrate the inhibitory effect of GBP2 on ECTV, and it offers insights into innovative antiviral strategies.

## 1. Introduction

Ectromelia virus (ECTV) is an obligate rodent virus, can infect laboratory mice and cause mousepox (also known as smallpox in mice) [1]. With the cessation of the global smallpox vaccination program in the late 1970s, a growing number of people worldwide are at risk of orthopoxvirus infections [2]. The emergence of human monkeypox in 2022 has prompted a renewed focus on poxviruses [3]. ECTV has been widely utilized as a research tool to investigate orthopoxvirus infections, providing valuable insights into viral immune evasion mechanisms and aiding in the development of antiviral drugs and vaccines [4]. Studies have shown that interferons are crucial in the antiviral response to ECTV infection [5]. While interferon does not directly attack pathogens, it triggers the expression of a network of genes, including ISGs [6]. GBPs are prominent ISGs and are vital in cell-autonomous immunity against bacterial, viral and protozoan pathogens [7]. GBPs belong to the dynamin GTPase superfamily and are mainly induced by type I interferons (IFN-α and IFN-β) and type II interferon (IFN-γ). Notably, IFN-γ demonstrates a higher efficacy in inducing GBPs compared to type I interferons. Other members of the dynamin GTPase superfamily include the immunity-related GTPases (IRGs, 45 kDa), myxovirus-resistant proteins (Mx, 70–80 kDa) and very large interferon-inducible GTPases (VLIG, 280 kDa). Among all the induced proteins by interferons, GBPs exhibit the highest expression, accounting for up to 20% of the total ISGs [8]. There are eleven identified GBP orthologs (mGBP1-mGBP11) in mice. The mouse GBP1, GBP2, GBP3, GBP5 and GBP7 genes are located on chromosome 3 (GBP*^chr3^*), the rest are on chromosome 5 (GBP*^chr5^*) [9]. GBP1 has been extensively researched due to its powerful antiviral, antiproliferative, and immune modulatory properties [10]. Recent research has demonstrated that GBP1 is effective against various viruses, including vesicular stomatitis virus (VSV) [11], hepatitis E virus (HEV) [12], hepatitis C virus (HCV) [13], and classical swine fever virus (CSFV) [14]. The antiviral activity of mGBP2 was initially reported in 2005, showcasing its capability to inhibit VSV and encephalomyocarditis virus (EMCV) [15]. mGBP2 has been found to display anti-murine norovirus (MNV) activity [16]. GBP2 and GBP5 work together to suppress the replication of HIV, influenza A virus (IAV), murine leukemia, Zika, measles and Marburg viruses [17]. GBPs can lyse pathogen-containing vacuoles, disrupting the niche of protozoa and bacteria, and can prevent the spread of the virions and target RNA viral replication complexes [18]. The antimicrobial effect of GBPs is also related to ubiquitination, autophagy and inflammasome formation [19]. Research on the mechanism of GBPs has mainly focused on their structure and enzymatic activity [20].

The structure of typical GBPs includes a conserved N-terminal globular large GTPase domain (LG) and a C-terminal α-helical domain (CTHD), which are connected by a hinge region [21,22]. The GTPase domain belongs to the nucleotide-dependent dimerization-activated G protein family. In the absence of nucleotide, it exists as a monomer, upon binding to guanosine triphosphate (GTP) analogs, it forms dimers via an LG-LG interface. The structural rearrangement of GBPs leads to the hydrolysis of GTP into guanosine diphosphate (GDP) and guanosine monophosphate (GMP) [23,24]. The energy released during nucleotide binding and hydrolysis results in changes in membrane conformational leading to fission or fusion, which in turn regulate cell growth and resistance to pathogen infection [25]. The CTHD domain is primarily involved in oligomerization and interaction with the plasma membrane [26]. GBP1, GBP2, and GBP5 contain a C-terminal end CaaX motif. This motif is important for isoprenylation. The nucleotide binding and hydrolysis process can manipulate the release of the “aaX” tail from the CTHD domains [27]. After the removal of “aaX”, the carboxyl group located at the end of “C” (cysteine) undergoes methylation, which enhances the ability of proteins to associate with endomembrane organelles. This process plays a crucial role in regulating the localization and trafficking of proteins [28,29].

Poxviruses have large DNA genomes (206–210 kbp) encoding approximately 200 proteins, 30–50% of the genes manipulate host innate and adaptive immune responses throughout the entire life cycle [30]. The interferon system is one of the most effective innate antiviral defense systems, and is countered by poxviruses through the evolution of immune response modifiers (IRMs). ECTV vIFN-γR can bind to IFN-γ and hinder its biological activity [31]. The EVM166 gene in the ECTV Moscow strain encodes a protein that can counteract the expression of type I IFNs [32]. For preventing the antiviral actions of ISGs, the vaccinia virus (VACV) encodes E3 protein to block the activation of PKR, OAS, and ISG15 [33,34]. As new antiviral ISGs are being identified, relatively few of them have been fully characterized. According to our laboratory’s transcriptome microarray analysis data, ECTV infection notably upregulates GBP1 and GBP2, indicating a potential impact on the virus [35]. Our study demonstrated that GBP2 plays critical role in restricting ECTV replication. These results provide a basis for future investigations into the intricate relationship between the poxvirus and the host’s innate immunity.

## 2. Materials and Methods

### 2.1. Cell Culture and Virus Infection

CV-1 cells (African green monkey kidney fibroblast cells) were cultured in RPMI 1640 basic medium (Gibco). BSR-T7 (derivative of baby hamster kidney cell), RAW264.7 (mouse macrophage) cells were grown in Dulbecco’s Modified Eagle Medium (DMEM; Gibco). The media was supplemented with 10% fetal bovine serum (Gibco) and 1% penicillin-streptomycin (Gibco). ECTV-Moscow (EVM, ATCC 1374) was propagated in CV-1 cells. The virus titers were determined by plaque assay. Briefly, prepare a 24-well plate of CV-1 cells. Create serial tenfold dilutions of the virus stock into serum-free medium and add 200 μL of the viral dilution to each well. Incubate the plate for 2 h. Then, overlay the wells with 1 mL of culture medium containing carboxy methyl cellulose (0.75%) and incubate at 37 °C for 5–7 days. To visualize the plaques, the cells were first fixed using 4% paraformaldehyde and then stained with 250 μL of crystal violet.

### 2.2. Antibodies

Primary antibodies against the following proteins or peptides were used: GBP2 (11854-1-AP, Proteintech, Wuhan, China); Flag (F3165, Sigma-Aldrich, Burlington, MA, USA); beta Tubulin (ab6046, Abcam, Waltham, MA, USA). Secondary antibodies used were as follows: HRP-conjugated goat anti-mouse IgG (SA00001-1, Proteintech, China); HRP-conjugated goat anti-rabbit IgG (SA00001-2, Proteintech, China). Polyclonal EVM H3L antiserum were generated in our laboratory. In brief, the EVM H3L target sequences were used to construct the pET30a-H3L recombinant prokaryotic expression plasmids. The plasmids were then transformed into Rosetta competent cells and induced with IPTG. Finally, antiserum was prepared by immunizing rabbits with the purified proteins.

### 2.3. Plasmids Construction and Transient Transfection

The full-length mouse GBP2, and truncated mouse GBP2 genes were extracted from IFN-γ stimulated RAW264.7 cells and cloned into the pcDNA3.1-Flag vector. Mutations were obtained by site-directed mutagenesis (D0206-QuickMutation Kit, Beyotime, Shanghai, China) from full-length GBP2-Flag tagged plasmids. The recombinant plasmids were transfected into Escherichia coli DH-5α competent cells (9057, Takara, Beijing, China). Single clones were selected for PCR identification, and the plasmids were extracted from the positive clone of Escherichia coli using the Endo-Free Plasmid Maxi kit (D6926, Omega Bio-Tek, Norcross, GA, USA). The plasmids were transfected into BSR-T7 cells using Lipofectamine 2000 (11668019, Invitrogen, Waltham, MA, USA) per the manufacturer’s instructions. Briefly, plasmid DNA and Lipofectamine 2000 were diluted in Opti-MEM (11058021, Invitrogen) at a mass-to-volume ratio of 1:2.5. A total of 1.5 μg of plasmid can generally be transfected in a 12-well plate. The mixture was incubated for 10 min and then added to the cells. After 16–24 h of transfection, cells were collected for western blot detection or were infected with ECTV. The primer sequences used for constructing the plasmids can be found in Supplementary Table S1.

### 2.4. shRNA-Mediated Knockdown of GBP2

GBP2 shRNA Lentiviral Particles (sc-41708-V, Santa Cruz, CA, USA) were utilized, which containing 3 target-specific constructs that encode 19–25 nt (plus hairpin) shRNA designed to knock down GBP2 expression in mouse cells. RAW264.7 cells were plated in a 12-well plate and when the cell confluency reached 50%, the media was replaced with 1 mL of media containing 5 μg/mL Polybrene. Subsequently, cells were infected by adding 20 μL of the lentiviral particles. The plate was gently swirled to mix and incubate overnight. The culture medium was then removed and replaced with 1 mL of complete medium without Polybrene. The cells were split at a ratio of 1:3 and incubated further for 24–48 h. Following this, puromycin (5 μg/mL) was used to select the cells. The medium was refreshed with puromycin-containing medium every 3–4 days until resistant colonies could be identified. Several colonies were picked, expanded, and assessed for stable shRNA expression. The non-target control shRNA Lentiviral Particles (sh-Con) (sc-108080, Santa Cruz, USA) were employed as negative controls.

### 2.5. qRT-PCR

Total RNA from stimulated cells was extracted using TRIzol reagent (15596018, Invitrogen) according to the manufacturer’s protocol. The isolated RNA was reverse transcribed into cDNA with PrimeScript RT reagent Kit (RR047A, Takara). qRT-PCR was performed using the primers listed in Supplementary Table S2. The relative expression levels of target genes were normalized to GAPDH and calculated using the threshold cycle (2^−ΔΔCt^) method.

### 2.6. Viral DNA Copy Number Test

To construct the standard plasmids, the amplified PCR products of EVM003 from ECTV-infected CV-1 cells were cloned into T-vector. Viral genomic DNA was extracted from the infected cell culture supernatants and whole-cell lysates. The EVM003 DNA copy number was measured using qPCR. A standard curve was established using the standard plasmids, and the viral DNA copy number was calculated through standard curve analysis.

### 2.7. Immunoblotting Analysis

Cells were lysed for 15 min on ice in cell lysis buffer, added 5 × SDS-loading buffer, boiled the sample for 10 min, then resolved by SDS-PAGE and transferred to PVDF membranes using a semi-dry transfer method. Incubate the membranes in 5% nonfat milk in Tris-buffered saline with 0.05% Tween 20 (TBST) for 1 h at room temperature or overnight at 4 °C. Primary antibody was incubated with the membranes at 4 °C overnight. Washed membrane 3 × 10 min with TBST, incubated with horseradish peroxidase-conjugated secondary antibody for 1 h at room temperature, washed membrane 3 × 10 with TBST, and protein bands were visualized with enhanced chemiluminescence reaction buffer (ECL) (34078, Thermo Fisher Scientific, Waltham, MA, USA) and imaged on a high-resolution image acquisition system (Bio-Rad). The grayscale values of the immunoblotting band were measured using Image Pro Plus software. The protein relative expression was calculated as the ratio of the gray value of the target protein to the gray value of the internal reference Tubulin. To normalize the data, the relative gray value of each set of proteins of interest was divided by the gray value of the control protein.

### 2.8. Cell Viability Assay

Cell viability was assessed using the Cell Counting Kit-8 (CCK-8) assay (GK10001, Glpbio). Cells were seeded at a density of 1 × 10*^4^* per well in 96-well plates. For sh-GBP2 and sh-Con RAW264.7 cells, they were cultured for 24 h. Subsequently, CCK-8 (10 μL/well) was added to each well, and the cells were incubated at 37 °C for 3 h. The absorbance at 450 nm was measured using a microplate reader. To examine the impact of plasmids on cell viability, BSR-T7 cells were transfected (1.5 μg/mL) when they reached 70–90% confluency and incubated for 24 h. CCK-8 detection was then performed following the same procedure as described above.

### 2.9. Statistical Analysis

The data in this paper are representative of three experiments and have been analyzed with the statistical methods described in the figure legends. All data are expressed as means ± SDs. Student’s *t* test, two-way ANOVA or one-way ANOVA were used in the statistical analysis, and significant differences are indicated in figures using the following symbols: * *p* < 0.1; ** *p* < 0.01; *** *p* < 0.001; **** *p* < 0.0001; “ns” is used to indicate no significance.

## 3. Results

### 3.1. Expression of GBP^chr3^ Is Strongly Upregulated upon ECTV Infection

**Figure 1:**
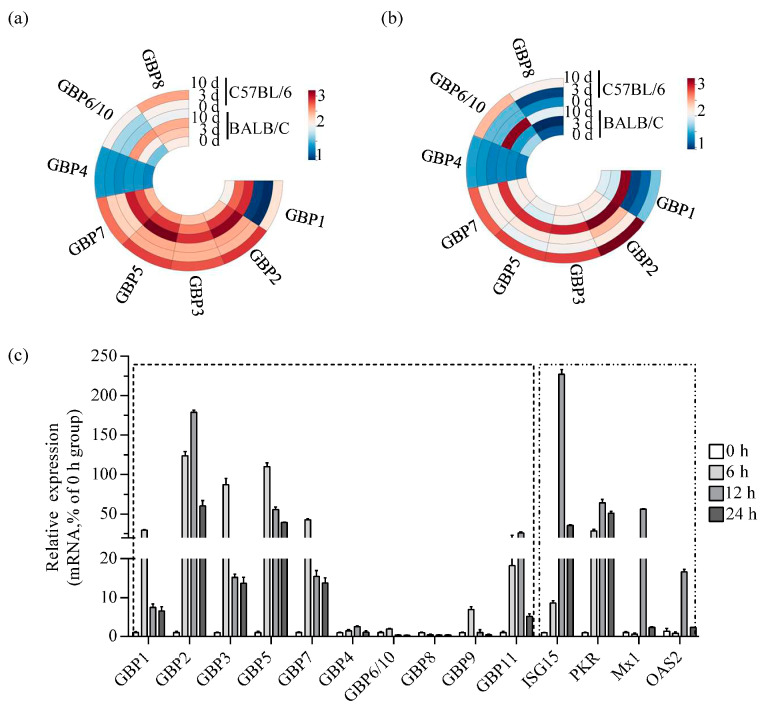
ECTV infection induces strong upregulation of GBP*^chr3^* expression in vitro and in vivo. (**a**,**b**) Heat map illustrates the expression levels of the GBP family proteins in mock-infected spleen (**a**) and blood (**b**) tissues versus those in ECTV-infected tissues at 3 and 10 dpi. The read values for individual transcripts at a given probe were transformed by log10 and constructed into a heat map using online ChiPlot software. (**c**) RAW264.7 cells were infected with ECTV-Moscow (MOI 1), and total RNA was extracted at 0, 6, 12 and 24 h post-infection respectively. GBP1 to GBP11, ISG15, PKR, Mx1, and OAS2 mRNA levels were analyzed by qRT-PCR (*n* = 3). GAPDH was used as a loading control. MOI, multiplicity of infection.

To provide a detailed analysis of the expression of GBP family members in ECTV-infected mouse tissues, a heat map was generated using transcriptome data that was made available by the laboratory online (https://www.ncbi.nlm.nih.gov/gds/, GSE100644 and GSE102850, accessed on 16 February 2018) [35]. It can be seen that most GBPs except GBP4 were strongly upregulated in BALB/c and C57BL/6 mice. Notably, the expression levels of genes located on chromosome 3 (GBP1, GBP2, GBP3, GBP5, and GBP7) were significantly higher than those on chromosome 5 (GBP4, GBP6/10, and GBP8). No expression of GBP9 and GBP11 was detected. The upregulation of GBPs was significantly higher in the infected versus non-infected group 10 days after infection (Figure 1a,b). The mRNA level ratio analysis indicated that the upregulation of GBP1 and GBP2 was significantly higher compared to other GBPs (Supplementary Table S3). However, the read value of GBP1 was lower than GBP2.

Macrophages are the initial inflammatory cells that respond to ECTV infection and are the primary producers of GBPs [36,37]. As such, they are an ideal model for examining the host’s innate immune response to ECTV. Transcript levels of GBPs were measured in RAW264.7 cells infected with ECTV. A significant increase in GBP2 mRNA levels in cells was observed, peaking at 12 h post-infection (hpi), showing an increase of over 180-fold. The mRNA levels of GBP1, GBP3, GBP5, and GBP7 were induced the most at 6 hpi, and upregulated approximately 30-fold, 90-fold, 110-fold, and 40-fold, respectively. The expression of GBPs on chromosome 5 is notably lower compared to the expression of GBPs on chromosome 3. In addition, the expression of ISG15, PKR, Mx1 and OAS2, which are primarily induced by type I interferon and have shown antiviral activity against poxviruses, was detected. The findings indicated that the levels of ISG15, PKR, Mx1 and OAS2 were up-regulated, with peak expression observed at 12 hpi. The fold increases were 235, 70, 60 and 20, respectively (Figure 1c). According to the in vivo and in vitro experiments, GBP2 were more strongly upregulated after ECTV infection, although not to the same extent as ISG15. This finding underscores its importance as candidate effector molecules in antiviral activity.

### 3.2. Overexpression of GBP2 Inhibits ECTV Replication

**Figure 2:**
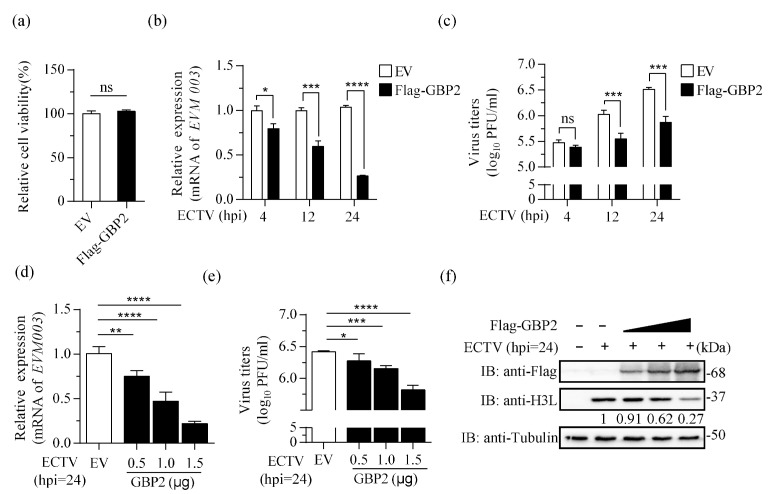
Overexpression of GBP2 inhibits ECTV replication. (**a**) The relative cell viability was measured using the CCK8 kit. (**b**,**c**) BSR-T7 cells were transfected with 1.5 μg/mL pcDNA3.1-Flag-GBP2 (Flag-GBP2) or the empty vector (EV) for 16 h. Afterwards, the cells were infected with ECTV (MOI = 1). At specified time points, the cell lysate and cell culture supernatant were harvested. The mRNA levels of EVM003 (**b**) and viral load (**c**) were measured using qRT-PCR and plaque assay, respectively. The viral mRNA relative expression, normalized to GAPDH, is presented as bar plots representing the mean ± S.D of technical repetition. (**d**–**f**) BSR-T7 cells were transfected with increasing doses of Flag-tagged GBP2 (0.5, 1.0, and 1.5 μg/mL) or the empty vector (1.5 μg/mL). The 0.5 and 1.0 μg/mL groups were supplemented with the empty vector to reach a final concentration of 1.5 μg/mL. After 16 h, cells were infected with ECTV (MOI = 1) for an additional 24 h. Samples were collected separately to determine the viral RNA transcripts (**d**), virus titer (**e**), and the expression of H3L protein (**f**). All three biological replicates showed similar results. * *p* < 0.1; ** *p* < 0.01; *** *p* < 0.001; **** *p* < 0.0001; ns, not significant.

The life cycle of ECTV in murine cells has not been fully elucidated, but is generally considered to last between 12 to 24 h. The genes are conventionally divided into early, intermediate and late classes based on their time of expression [38]. To investigate the effect of overexpressing GBP2 on ECTV (MOI = 1), the transcription of EVM003, which encodes the only active viral tumor necrosis factor receptor (vTNFR), was detected at 4, 12, and 24 hpi. EVM003 possesses transcriptional regulatory signals consistent with early and late expression in the infection cycle [39]. The results showed that the EVM003 mRNA levels were reduced in the presence of GBP2 at every stage of viral replication (Figure 2b). Subsequent plaque experiments demonstrated that high expression of GBP2 can inhibit the proliferation of ECTV at both 12 hpi and 24 hpi, but it had no effect at 4 hpi (Figure 2c). By gradually increasing the expression of GBP2, it was observed that GBP2 effectively inhibits ECTV replication by suppressing viral transcription, protein expression, and viral load in a dose-dependent manner (Figure 2d–f). Moreover, the overexpression of GBP2 did not have any adverse effects on cell viability, as confirmed by the CCK8 assay (Figure 2a). These findings suggest that GBP2 exhibits a dose-dependent hindrance to ECTV replication, and the inhibition step may occur in the late stage of viral replication.

### 3.3. Knockdown of Endogenous GBP2 Enhances ECTV Replication

**Figure 3:**
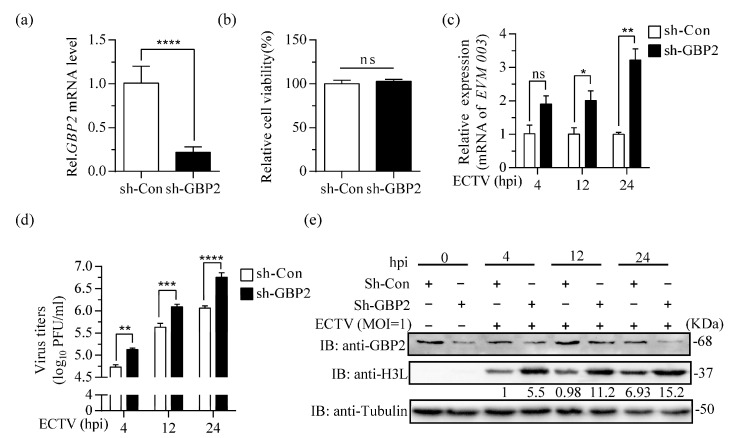
Knockdown of GBP2 enhances ECTV replication. (**a**) qRT-PCR analysis of GBP2 knockdown in sh-GBP2 and negative control (sh-Con) screening RAW264.7 cells. GAPDH was used as a loading control. (**b**) CCK8 tests of relative cell viability in sh-GBP2 and sh-Con screening RAW264.7 cells. (**c**–**e**) sh-GBP2 or sh-Con RAW264.7 cells were infected with ECTV at a MOI of 1 for 4, 12 and 24 h. The levels of viral mRNA, virus titers, and viral H3L protein expression were determined by RT-qPCR (*n* = 3) (**c**), plaque assay (*n* = 4) (**d**), and immunoblotting (**e**), respectively. Immunoblotting was performed to analyze cell lysates using antibodies against viral H3L protein and Tubulin. GBP2 antibody was used to detect the expression of endogenous GBP2 in RAW264.7 cells. * *p* < 0.1; ** *p* < 0.01; *** *p* < 0.001; **** *p* < 0.0001; ns, not significant.

To confirm the antiviral effect of GBP2, we utilized a lentivirus system with puromycin resistance characteristics to knockdown GBP2 expression in RAW264.7 cells. The successful knockdown of GBP2 was confirmed through RT-qPCR results (Figure 3a). Subsequently, both GBP2 knockdown RAW264.7 cells (sh-GBP2) and control RAW264.7 cells (sh-Con) were infected with ECTV (MOI = 1). The results revealed a significant increase in viral transcription, virus titer, and protein level at 12 h and 24 h. Besides, the presence of elevated levels of viral replication was observed as early as 4 h after infection, indicating that GBP2 plays a vital role in restricting the growth of ECTV (Figure 3b–d).

### 3.4. Truncated N-Terminal LG Domain of GBP2 Impairs Its Antiviral Effect

**Figure 4:**
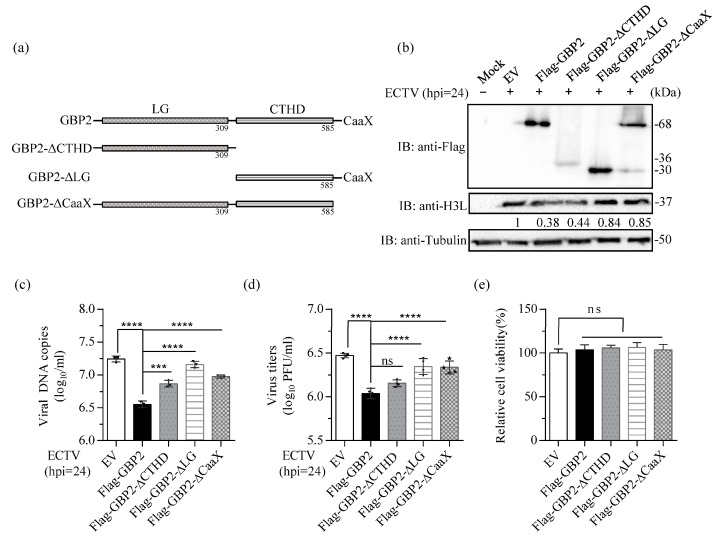
The GTPase domain of GBP2 is essential for its antiviral effect. (**a**) Schematic presentation of full-length and truncated domain of GBP2. (**b**–**d**) BSR-T7 cells were transfected with Flag-tagged full-length and the three truncated GBP2 plasmids (1.5 μg/mL) for 16 h, respectively, the empty vector (EV) plasmid (1.5 μg/mL) was used as control, then infected with ECTV at a MOI of 1 for 24 h. The cells were collected separately for H3L protein expression (**b**), viral DNA copies (**c**), and virus titers analysis (**d**). The gray value in (**b**) were normalized to the EV control (set as 1). (**e**) The relative cell viability was measured using the CCK8 kit. *** *p* < 0.001; **** *p* < 0.0001; ns, not significant.

GBPs utilize their various functional domains to combat diverse pathogens. The GTPase activity is crucial for GBP2-mediated immune response against MNV-1 and is antagonized by the viral protein NS7 [16]. Additionally, GBP2 inhibits EMCV through its GTP binding activity [15]. Studies have demonstrated that GBP1 exerts an antiviral effect against HCV and CSFV by utilizing its GTPase activity [13,14]. But the GTPase-defective GBP5 maintains its antiviral ability against respiratory syncytial virus (RSV) and HIV. Conversely, C-truncated GBP5 which impair its Golgi apparatus localization can disrupt the inhibitive activity [40,41]. To investigate the specific domains of GBP2 that impede ECTV replication, we generated three variants of GBP2 based on online Unified Protein Database prediction: Flag-tagged truncated LG domain (GBP2-ΔLG), Flag-tagged truncated CTHD (GBP2-ΔCTHD), and a variant with the CaaX motif deleted (GBP2-ΔCaaX) (Figure 4a). BSR-T7 cells were used to overexpress these recombinant plasmids and were then infected with ECTV to determine the impact of the domains. According to our findings, the removal of the LG domain in GBP2 led to a loss of its ability to inhibit virus replication. On the other hand, GBP2-ΔCTHD, still exhibited antiviral activity. It is worth noting that the effectiveness of GBP2-ΔCaaX in inhibiting viral propagation and protein expression was significantly reduced (Figure 4b–d). Generally, the GTPase domain of GBP2 is indispensable in limiting ECTV replication. On the other hand, the prenylation-defective GBP2-ΔCaaX also affect ECTV inhibition, indicating that the perinuclear membranes-targeting is of great significance for its antiviral effect.

### 3.5. The Lys-51 at the N-Terminal of GBP2 Is the Key Site for ECTV Inhibition

**Figure 5:**
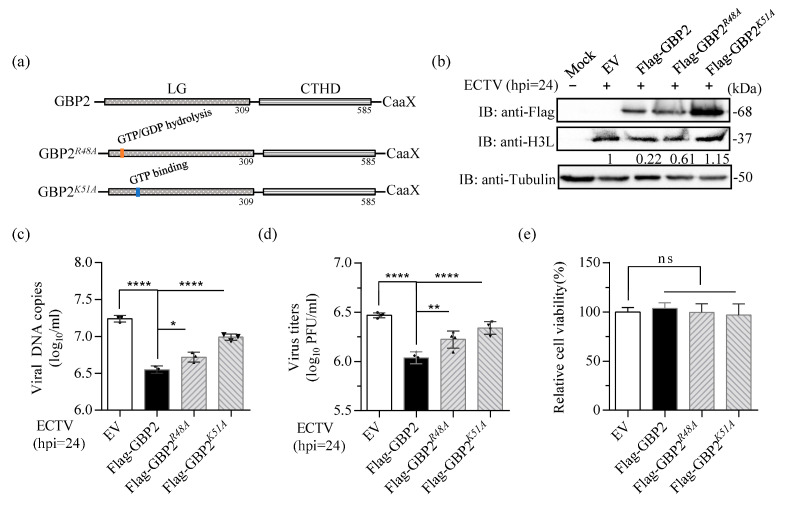
The K51 residue of GBP2 is the key site for anti-ECTV activity. (**a**) Schematic presentation of the full-length and the two mutants of GBP2. (**b**–**d**) BSR-T7 cells were transfected with either full-length or mutant plasmids of GBP2, while an empty vector served as the control (EV). The cells were then infected with ECTV (MOI = 1) for 24 h. Separate collections of cells were used for immunoblotting (**b**), qPCR (*n* = 3) (**c**), and plaque assay (*n* = 4) (**d**) analysis. The gray value in (**b**) were normalized to the EV control (set as 1). (**e**) The relative cell viability was measured using the CCK8 kit. * *p* < 0.1; ** *p* < 0.01; **** *p* < 0.0001; ns, not significant.

Previous studies have demonstrated the significance of Arg-48 (R48) and Lys-51 (K51) residues in the GTPase activity of GBP1, GBP2, and GBP5 [13,22,39]. The R48 and K51 residues within the LG domain are critical for GBP2-mediated anti-MNV-1 activity [16]. GBP1*^K51A^* mutation was unable to inhibit IAV replication [42], and the K51 of GBP1 is crucial for the inhibition of CSFV replication [14]. Hence, we created Flag-tagged mutants GBP2*^R48A^* and GBP2*^K51A^* to investigate the impact of mutant R48 and K51 of GBP2 on its antiviral effect on ECTV (Figure 5a). The two mutant plasmids were overexpressed, and the results revealed that the mutant GBP2*^K51A^*, which is defective in GTP binding, completely lost the ability to limit EVM003 amplification, the virus proliferation and protein expression. Conversely, the mutant GBP2*^R48A^*, which has a defect in GTP hydrolysis, only faintly reduced the effect on ECTV restriction (Figure 5b–d). Together, these results reveal that K51 of GBP2 is essential for its antiviral activity, which signify that the GTP-binding capacity is indispensable for GBP2 to limit ECTV replication.

## 4. Discussion

The ongoing transmission of monkeypox from human-to-human raises concerns about potential epidemics in areas with low or no immunity to orthopoxviruses. Therefore, it is imperative to investigate the antiviral strategies against monkeypox virus and its closely related variola virus. While the existence of the GBP protein was discovered in 1979, its antiviral activity was not confirmed until 20 years later through research on VSV and EMCV [43]. In this study, we present data demonstrating that GBP2 exhibits significant inhibitory activity to ECTV, expanding our understanding of the antiviral activity of GBP family members against large DNA viruses. It is important to note that we assessed the effect of GBP2 on ECTV using a plaque assay, and observed a difference in viral titer of approximately 0.5 log. Previous research has shown that even a modest reduction of 1–2 log reduction in MPXV can substantially decrease morbidity and mortality in non-human primates [44]. Thus, GBP2 thus had a mild yet statistically significant and reproducible inhibitory effect on the growth of ECTV.

It has been reported that MxA, another member of the GTPase family, exhibits inhibitory effects on two large DNA viruses, namely African swine fever virus (ASFV) and MPXV [45,46]. Remarkably, the inhibition of ASFV replication was linked to the recruitment of the MxA protein to the perinuclear viral assembly site, which encompasses the viral factory [45]. However, it does not show any activity against vaccinia virus (VACV) and cowpox virus (CPXV) [47]. This difference could possibly be attributed to genetic variations between MPXV and other orthopoxviruses, as well as the specific cell lines and clones used in these studies. To gain a more comprehensive understanding of the inhibitory activity of GBPs in different cellular virus systems, further investigations with additional cell lines and related viruses are warranted. The impact of antiviral molecules is typically more significant in primary cells or in vivo. When overexpression of GBP2 was utilized to investigate its impact on ECTV, it predominantly exhibited its influence during the later phase of infection (Figure 2). Conversely, when knockdown of GBP2 was employed, it exerted a notable enhancing effect in both the early and late stages (Figure 3). This further elucidates that the expression level of GBP2 in different cells directly influences its efficacy against ECTV. GBP is a gene that is induced by interferon expression. However, in the screened knockdown cells and control RAW cell lines, GBP2 expression can also be observed even in the absence of ECTV infection. When comparing the protein levels after infection with the control, no significant up-regulation is observed. This suggests that lentivirus infection may have an impact on GBP expression, but further investigation is needed to confirm this. On the other hand, according to our analysis of transcript levels after ECTV infection of RAW264.7 cells, we observed a sharp decrease in GBP transcription 6–12 h after infection (Figure 1c). This suggests that ECTV may encode a gene against GBP production, or it could be that there is a process of translation inhibition or rapid protein degradation to prevent aggregation or cellular toxicity. The co-evolution between hosts and pathogens is driven by an ongoing arms race. Poxviruses encode a large number of genes for self-replication and to hijack or evade host immune responses. The detailed mechanism is currently being studied.

The deletion of the N-terminal LG domain of GBP2 led to the complete loss of its inhibition on ECTV. This provides further confirmation of the antiviral effect of GBP2 on ECTV. Interestingly, although the inhibitory function is diminished when the CaaX tail is removed, GBP2-ΔCTHD, which also lacks the CaaX motif, still retains its antiviral ability (Figure 4b–d). Previous research has demonstrated that a splice variant of hGBP3 (hGBP3-ΔC) with a modified C-terminal α-helical domain effectively resists influenza infection, unlike the full-length GBP3 [48]. Since most GBPs exert their effects through the large GTPase domain, it is speculated that the C-terminal α-helical domain may generally inhibit this activity. It is suggested that the targeting of CaaX to the endomembrane organelles could regulate the inhibition of GTPase activity by the C-terminus α-helical domain. Further investigation is required to gain a better understanding of this role.

The GTPases domain encodes four conserved motifs: phosphate-binding loop (p-loop, G1), switch I (G2), phosphate- and Mg^2+^-binding switch II (G3), and nucleotide-specificity providing motif (G4) [22]. G1 has been the most extensively studied since it reacts with the phosphate groups of nucleotides and is rich in glycine. The glycine-rich region shares similarities with the nuclear localization system, which is involved in various biological processes such as cell division, signal transduction, ribosome assembly, and protein synthesis. Severe loss of GTPase activity occurs due to mutations in G1 lysine or arginine residues. This is because Lys51(K51) interacts with the β and γ-phosphate group of the bound GTP, and a mutation of K51 in the G1 motif results in the formation of a polar bond with Thr98 of G3, leading to the dysfunctional of the protein [20]. R48 is highly conserved and acts as a catalytic arginine finger, reorienting the nucleotide towards the reaction center in the dimerization GTPase domain. It stabilizes the negative charge accumulated in the hydrolysis transition state. The R48 mutation reduces GTP hydrolysis activity [25]. The affinity of the R48A mutant of mGBP2 for GMP remains within the range of the WT protein. However, in hGBP1, the corresponding mutant exhibits a 15-fold decrease in affinity compared to the WT [49]. Our findings indicate that the K51 mutation significantly decreases the inhibitory capacity of GBP2, highlighting the importance of the ability to bind GTP analogs for GBP2 (Figure 5b–d). This shows that GBP2 exerts antiviral effect mainly through its GTPase activity.

GBPs are primarily located in the cytoplasm of both immune and nonimmune cells, with GBP2 specifically associated with the perinuclear membrane compartments [50]. Interestingly, poxviruses have a unique replication process that occurs exclusively within cytoplasmic viral factories located near the nucleus [51]. GBPs are known to play a crucial role in clearing intracellular pathogens by inducing lysis of pathogen-containing vacuoles. However, it is currently unknown whether GBPs perform their function by lysing the virus factory. In this study, our focus was primarily on assessing the effectiveness of GBP2 against ECTV as a justification for its further development as a therapeutic against highly pathogenic orthopoxviruses. This study focused solely on the effect of GBP2 on ECTV. The antiviral activity of other GBPs on ECTV and the coordination between these GBP members have not been explored in this study. Further investigation is needed to understand these aspects. 

This study presents new findings on the anti-ECTV activity of GBP2. These results offer potential targets for antiviral interventions.

## 5. Conclusions

Guanylate-binding protein 2 (GBP2) exerts inhibitory effects on the replication of ectromelia virus (ECTV) through its GTPase activity. The crucial site for ECTV inhibition is Lys-51 located at the N-terminal of GBP2. This study contributes to the understanding of the role of GBP in countering DNA viruses. The findings presented here serve as a basis for further investigation into the intricate interplay between poxviruses and the innate immunity of the host. Additionally, these results offer insights for future studies on the development of antiviral drugs.

## Data Availability

All data are reported in the manuscript.

## Supplementary Materials

The following supporting information can be downloaded at: https://www.mdpi.com/article/10.3390/microorganisms11092258/s1, Table S1: Primer sequences for plasmids construction in this study; Table S2: Primer sequences for qRT-PCR in this study; Table S3: Fold-changes of normalized expression in GBPs probes.
