# Supplementary material for: Guanylate-Binding Protein 2 Exerts GTPase-Dependent Anti-Ectromelia Virus Effect
Source: Microorganisms. 2023 Sep 8;11(9):2258. doi: 10.3390/microorganisms11092258 (PMC10534507; doi:10.3390/microorganisms11092258)
Supplement: Supplementary file 1 [file microorganisms-11-02258-s001.zip › microorganisms-2581557-supplementary.pdf]

Supplementary Materials

Table S1 Primer sequences for plasmids construction in this study

Primer	Sequence (5'–3')	Note
Flag-GBP2-F	cggggtaccATGGCCTCAGAGATCCACATG	KpnI
Flag-GBP2-R	gctctagaAGTCAGAGTATAGTGCACTTCCCAG	XbaI
Flag-GBP2-ΔCTHD-F	cggggtaccATGGCCTCAGAGATCCACATG	KpnI
Flag-GBP2-ΔCTHD-R	gctctagaagGCAGGGGAGAGACCCATTGCTG	XbaI
Flag-GBP2-ΔLG-F	cggggtaccATGGAGAGTGCTGTGCTGAC	KpnI
Flag-GBP2-ΔLG-R	gctctagaagTCAGAGTATAGTGCACTTCCCAG	XbaI
Flag-GBP2-ΔCaaX-F	cggggtaccATGGCCTCAGAGATCCACATG	KpnI
Flag-GBP2-ΔCaaX-R	gctctagaagCTTCCCAGACGATTTGTTTTGC	XbaI
Flag-GBP2-R48A-F	CAATCGTGGGCCTCTAC**GC**CACAGGCAAATCCTACCTGATGAA	base142/1
Flag-GBP2-R48A-R	TTCATCAGGTAGGATTTGCCTGTGGCGTAGAGGCCCACGATTG	43 CG-GC
Flag-GBP2-K51A-F	GGCCTCTACCGCACAGGCA**GC**TCCTACCTGATGAACAAGCTAG	base151/1
Flag-GBP2-K51A-R	CTAGCTTGTTCATCAGGTAGGAGCTGCCTGTGCGGTAGAGGCC	52 AA-GC

Table S2 Primer sequences for qRT-PCR in this study

Primer	Sequence (5'-3')	Note
qGAPDH-F	AGGTCGGTGTGAACGGATTTG	qRT-PCR for
qGAPDH-R	TGTAGACCATGTAGTTGAGGTCA	detection of GAPDH
qGBP1-F	AAACCAGGAGGCTACTACCTTTTT	qRT-PCR for
qGBP1-R	GTATTTTCTCAGCATCACTTCAGC	detection of GBP1
qGBP2-F	CTGCACTATGTGACGGAGCTA	qRT-PCR for
qGBP2-R	GAGTCCACACAAAGGTTGGAAA	detection of GBP2

qGBP3-F	CCAGAAAACCAACTGGAACGGAA	qRT-PCR for
qGBP3-R	TCTCCAGACAAGGCACAGTC	detection of GBP3
qGBP5-F	AGCTGAAGCAAGGTAGCGAT	qRT-PCR for
qGBP5-R	CGTTGCTGAGTGTTGGAAGC	detection of GBP5
qGBP7-F	TTGAGGAAATGCCAGAGGACCAGT	qRT-PCR for
qGBP7-R	GTCTCCACTATTGATAGCATCCACG	detection of GBP7
qGBP4-F	GCCAAGATCAAGACCCTCAG	qRT-PCR for
qGBP4-R	CCACGTAGGTTGTCACCAGA	detection of GBP4
qGBP6/10-F	AGTGATGACTACCTGGAGAAT	qRT-PCR for
qGBP6/10-R	AGAATCAAGTTTTTGTAAAAGTTC	detection of GBP6/10
qGBP8-F	GCATGCCACACCCCACTAAAC	qRT-PCR for
qGBP8-R	GAAGCACACTGAGGGCAAAGAT	detection of GBP8
qGBP9-F	TTCCAAAACTTTCTCCAGTCACAGTA	qRT-PCR for
qGBP9-R	GGCACGCTCCTCTGCAA	detection of GBP9
qGBP11-F	AAGCTGAAGTTAAATGGGGAAG	qRT-PCR for
qGBP11-R	CACTTCCGATTTGGAAAGAAAC	detection of GBP11
qISG15-F	GGTGTCCGTGACTAACTCCAT	qRT-PCR for
qISG15-R	CTGTAISG15CCACTAGCATCACTGTG	detection of ISG15
qMx1-F	GACCATAGGGGTCTTGACCAA	qRT-PCR for
qMx1-R	AGACTTGCTCTTTCTGAAAAGCC	detection of Mx
qOAS2-F	TTGAAGAGGAATACATGCGGAAG	qRT-PCR for
qOAS2-R	GGGTCTGCATTACTGGCACTT	detection of OAS2
qPKR-F	ATGCACGGAGTAGCCATTACG	qRT-PCR for
qPKR-R	TGACAATCCACCTTGTTTTCGT	detection of PKR
qEVM003-F	TCTGTCCTTTAACAGCATAGATGTAGA	qRT-PCR for
qEVM003-R	TGTTAACTCGGAAGTTGATATGGTA	detection of EVM003

Table S3 Fold-changes of normalized expression in GBPs probes

Tissue	Spleen				Blood			
Mouse	Infection to mock ratio							
strain	BABL/c		C57BL/6		BABL/c		C57BL/6	
gene	3 d	10 d	3 d	10 d	3 d	10 d	3 d	10 d
GBP1	4.3152	6.5993	0.8588	17.8440	0.8522	15.5504	1.3855	3.8967
GBP2	3.5985	6.3365	0.8846	2.9842	0.7788	10.6632	0.5429	5.9683
GBP3	1.8218	3.2666	0.9770	2.0966	0.8803	6.4064	0.8338	5.0167
GBP5	2.2365	4.5931	0.9460	2.2284	0.7307	6.9858	0.6427	4.5712
GBP7	2.3937	3.6124	1.0377	2.4435	0.8711	5.4828	1.0133	4.2548
GBP4	1.1986	1.1994	1.1562	1.1766	1.1510	1.1787	1.2363	1.4026
GBP6/10	3.3628	9.4238	1.0805	2.6503	0.4898	26.4251	1.0109	7.5019
GBP8	1.6878	2.2422	1.2085	3.2046	0.6824	7.1804	0.5045	6.0624
